# Supplementary material for: The role of gestures in autobiographical memory
Source: PLoS One. 2023 Feb 24;18(2):e0281748. doi: 10.1371/journal.pone.0281748 (PMC9955584; doi:10.1371/journal.pone.0281748)
Supplement: Attachment — Submitted filename: Response to Reviewer Comments_2.docx [file pone.0281748.s003.docx]

*Dear Editor,*

*We thank you for this opportunity to improve on the quality of our manuscript based on the rich set of comments provided by the Editor and the Reviewers. Below, we provide detailed point-by-point responses (italicized) to these comments.*

Editor: “There are problems with spacing ( gaps were there should be none) and a spelling error on p. 9 indeces should be indices. Can you carefully check these points and resubmit.”

***RESPONSE:*** *Thank you. We have addressed all of these points in the submitted revised version.*
