# Supplementary material for: The role of gestures in autobiographical memory
Source: PLoS One. 2023 Feb 24;18(2):e0281748. doi: 10.1371/journal.pone.0281748 (PMC9955584; doi:10.1371/journal.pone.0281748)
Supplement: Attachment — Submitted filename: Response to Reviewer Comments.docx [file pone.0281748.s002.docx]

*Dear Editor,*

*We thank you for this opportunity to improve on the quality of our manuscript based on the rich set of comments provided by the Editor and the Reviewers. Below, we provide detailed point-by-point responses (italicized) to these comments.*

p. 3, paragraph 2, penultimate sentence: it's not clear from the wording how deictic gestures are classified; reword for clarity.

***RESPONSE:*** *Thank you. The text is changed. Please see below:*

*“deictic gestures include the type of hand movements that involve pointing or reaching to the objects or abstract entities (McNeill, 1992).”*

p. 10, fifth line from the bottom: could you briefly characterize what a "flag ceremony" is? If these were American participants, it would probably mean swearing allegiance. But I suspect that this flag ceremony involves some different activities?

***RESPONSE:*** *We changed the name to “Flag-raising morning ceremony”. We added a note explaining what it entails. Please see below:*

*“... a typical flag raising morning ceremony in school, which is a common practice in some countries where students gather in the school yard to sing the national anthem together.”*

p. 13, line 3 from the bottom: how were word tokens calculated? Notably were false starts and self-repetitions included or not? (I don't care if they were or not; I just want the specification so that future researchers can do the same!)

***RESPONSE:*** *The text below was added to the main text.*

*“Memory narratives were transcribed verbatim from the video recordings with all utterances including false starts, fillers, and other disfluencies in addition to words.”*

p. 14, Figure 1: the label of the x-axis says "gestures per utterance" but the text says that it was gestures per word that was calculated. If it was utterances, make sure that "utterance" is defined.

***RESPONSE:*** *This is fixed as utterance throughout the text where relevant.* *In page 12, we note that “....with all utterances including false starts, fillers, and other disfluencies in addition to words.”*

p. 14, first line of text: exactly what analysis was done?

***RESPONSE:*** *Kendall’s Tau rank correlation analysis. This is noted in the Analytical Plan section.*

p. 14, penultimate line: I will simply register my disagreement here with the authors' decision to throw out the zeros. If they think that gesturing leads to the inclusion of more episodic details, then not gesturing at all should mean the inclusion of few episodic details.

***RESPONSE:*** *The short answer to this comment is: Yes, we agree with the Reviewer and did not throw out the zeros. Please see the longer response below:*

*“Our original reasoning was that not including individuals who did not gesture would make the conclusions stronger (i.e., gestures lead to episodic content). This had been discussed quite extensively within the research team. We ended up including the participants who did not gesture. However, much to our embarrassment, we forgot to remove the portion of the text that described our reasons for not including them. Thus, the numbers in the first submission actually reflect the full set of participants, including the ones who did not gesture. We now remove that bit of the text in the revised version.*

*For comparison reasons, and for the reviewer’s attention, we re-ran the analyses without those participants who did not gesture during the narrative.* ***Item A*** *below shows those results, and* ***item B*** *shows the full set. As you can see the numbers only slightly differ from each other.*

A. *For the past events, representational gestures correlated with internal details (****τ = .33****, p = .005). To delve into further examination of which particular episodic details were related to the representational gestures, we looked into internal detail categories. Representational gestures were correlated with internal place details (****τ = .30****, p = .02) as well as internal perceptual details (****τ = .38****, p = .002), event details (τ = .23, p = .048).*
B. *For the past events, representational gestures correlated with internal details (****τ = .27****, p =.02). To delve into further examination of which episodic details were related to the representational gestures, we looked into internal detail categories. Representational gestures were correlated with internal place details (****τ = .28****, p = .02) as well as internal perceptual details (****τ = .36****, p = .002) as depicted in Table 2.*

*p. 14, last line, "utterance": utterance or word??????*

***RESPONSE***: *It should be “utterance”.*

p. 15, Table 1: consider putting # or N in front of the gesture lines to make it clear that this is not the gesture rate (i.e., per word or per utterance, whichever way it was actually done!).

***RESPONSE:*** *We added a note under Table 1 to indicate that the values are, in fact, counts.*

p. 21, end of first paragraph: here I was wondering whether the representational gesture rate and non-representational gesture rate was correlated within a task? It might just be that it is the 'gesturiness' of a person that predicts the episodic details…

***RESPONSE:*** *Yes, as presented in Tables 2 & 3, they are correlated. However, it was only in the past event condition, we were able to spot a relationship between episodic details and representational gestures. We did not observe non-representational gestures to be associated with episodic details in any condition.*

*Interestingly though, non-representational gesture use was also correlated with the subjective sense of recollection. Although prior work has not tested this relationship, the evidence on gestures alleviating cognitive load in narrative recall and problem-solving (e.g., Cook et al., 2010; Lin, 2021; Overoye & Wilson, 2020; Ping & Golden-Meadow, 2010) suggests that reduced cognitive demands during retrieval via the use of gestures may lead to richer reflective recollective experience. This is added in the Discussion.*

***Additional changes:***

*Although it was not raised by the reviewers and the editor, we realized mismatches when reporting the statistics in the Results sections. Thus, we revised this section by italicizing the "p" denotation via track-changes.*

*We also updated the references section for the added and removed citations in the revised version of the paper.*
